# Preclinical Evaluation of a Food-Derived Functional Ingredient to Address Skeletal Muscle Atrophy

**DOI:** 10.3390/nu12082274

**Published:** 2020-07-29

**Authors:** Roi Cal, Heidi Davis, Alish Kerr, Audrey Wall, Brendan Molloy, Sweeny Chauhan, Sanja Trajkovic, Ian Holyer, Alessandro Adelfio, Nora Khaldi

**Affiliations:** Nuritas Limited, Dublin D02 RY95, Ireland; cal.roi@nuritas.com (R.C.); davis.heidi@Nuritas.com (H.D.); kerr.alish@nuritas.com (A.K.); molly.brendan@Nuritas.com (B.M.); chauhan.sweeny@Nuritas.com (S.C.); trajkovic.sanja@Nuritas.com (S.T.); holyer.ian@nuritas.com (I.H.); Adelfio.Alessandro@Nuritas.com (A.A.); Nora@Nuritas.com (N.K.)

**Keywords:** protein synthesis, muscle atrophy, inflammation, skeletal muscle, aging, immobilization, peptide, bioactive, functional ingredient

## Abstract

Skeletal muscle is the metabolic powerhouse of the body, however, dysregulation of the mechanisms involved in skeletal muscle mass maintenance can have devastating effects leading to many metabolic and physiological diseases. The lack of effective solutions makes finding a validated nutritional intervention an urgent unmet medical need. In vitro testing in murine skeletal muscle cells and human macrophages was carried out to determine the effect of a hydrolysate derived from *vicia faba* (PeptiStrong: NPN_1) against phosphorylated S6, atrophy gene expression, and tumour necrosis factor alpha (TNF-α) secretion, respectively. Finally, the efficacy of NPN_1 on attenuating muscle waste in vivo was assessed in an atrophy murine model. Treatment of NPN_1 significantly increased the phosphorylation of S6, downregulated muscle atrophy related genes, and reduced lipopolysaccharide-induced TNF-α release in vitro. In a disuse atrophy murine model, following 18 days of NPN_1 treatment, mice exhibited a significant attenuation of muscle loss in the soleus muscle and increased the integrated expression of Type I and Type IIa fibres. At the RNA level, a significant upregulation of protein synthesis-related genes was observed in the soleus muscle following NPN_1 treatment. In vitro and preclinical results suggest that NPN_1 is an effective bioactive ingredient with great potential to prolong muscle health.

## 1. Introduction

Skeletal muscle accounts for approximately 40% of total human body weight and for 30–50% of whole-body protein turnover [1]. Its critical role in many metabolism and molecular disease-related pathways makes the preservation of the muscle mass of paramount importance [2]. Skeletal muscle health is regulated by a complex multifactorial process aimed at maintaining the balance between muscle protein synthesis (MPS) and breakdown (MPB) [3]. Dysregulation of the mechanisms involved in skeletal muscle mass maintenance can result in significant muscle wasting as seen in aging (sarcopenia) and disease (cachexia) or low activity associated with sedentary lifestyles and immobilization [3,4]. Additionally, a prolonged period of muscle wasting can adversely impact insulin sensitivity, metabolism, and fat mass in an otherwise healthy population [5]. The ability to modulate muscle metabolism not only addresses atrophy associated with such muscle disorders but also presents an opportunity for maintaining, building, or prolonging muscle mass health in the general population.

The mammalian target of rapamycin (mTOR) is a master regulator of the anabolic machinery for protein translation initiation in the cell [6]. The maintenance of skeletal muscle mass and function is specifically regulated by mTOR complex 1 (mTORC1) signaling through its main downstream effectors, ribosomal protein S6 protein kinase 1 (S6K1), and eukaryotic initiation factor 4E binding protein 1 (4EBP1) [7]. Muscle catabolism, on the other hand, is mainly modulated by the ubiquitin proteasome pathway (UPP), which in healthy conditions keeps the turnover of proteins and helps maintain protein homeostasis levels [8]. The UPP activity is markedly promoted in muscle waste conditions by transcriptional activation of two crucial muscle-specific ubiquitin ligases, muscle atrophy F-box (MAFbx) and muscle RING finger 1 (MuRF-1) [9,10].

Many current studies examining nutritional interventions for muscle health focus solely on the muscle, ignoring the potential cross talk between other tissues and cells i.e. immune cells [5]. Systemic inflammation has been pointed out as one of the primary causes that leads to muscle loss [11]. Several reports suggest that the inflammation master regulator, Nuclear Factor kappa-light-chain-enhancer of activated B (NF-κB), is capable of inducing muscle loss through the increased expression of the muscle ubiquitin ligase MuRF-1 [11,12]. Importantly, NF-κB-regulated proinflammatory cytokines such as tumour necrosis factor alpha (TNF-α) and interleukin-6 (IL-6) are the most important inducers of muscle waste, particularly in chronic conditions like sarcopenia [13,14]. Similarly, in healthy patients, TNF-α infusion has shown to increase protein degradation [15]. This is also the case in individuals with cancer cachexia, chronic obstructive pulmonary disease (COPD), and disuse who show elevated serum TNF-α levels which correlate with muscle loss [16,17].

Existing solutions to prevent muscle waste focus mainly on mTOR activation via the supply of nutrition, e.g., amino acids, especially leucine, but recent results suggest that fortification of meals with leucine could be not as effective as it was thought, as leucine has been shown to not attenuate the decline of muscle mass and strength during a seven-day limb immobilization intervention with young men [18]. As healthy muscle function is involved in numerous critical activities, it affects a wide spectrum of individuals worldwide. Therefore, any nutritional intervention identified must work to promote good muscle health in a broad population [14].

Peptides are protein fragments that can have a positive impact on bodily functions through specific protein-protein interactions. Bioactive peptides are known to modulate the digestive, endocrine, cardiovascular, immune, and nervous systems [19,20]. Exogenous bioactive peptides present in hydrolyzed protein sources offer the scientifically intriguing concept that compounds latent in food can exert functional effects beyond that of the purely nutritional [21,22]. Therefore, they present a prime opportunity for prevention and treatment of chronic disorders, including muscle atrophy [23,24], however they first must be unlocked from the proteome to confer bioactivity. It is more and more recognized that health benefits are associated with plant-based dietary protein sources [25] and a plant-based diet is increasingly seen as a potential approach to address sarcopenia in an aging population [26]. While whole and raw legumes are noteworthy sources of nutrition [27], so far there has been little success in addressing muscle mass maintenance with reasons cited such as low digestibility [26]. Despite these findings, functional activities have been associated with legumes, including antifungal, anticancer, and antidiabetic activities being specifically associated to *V. faba* [28,29,30]. Additionally, there has been a recent research effort to use a blended legume enriched food to investigate anabolic properties in an aged rat model [31]. By using this legume blend including *V. faba,* digestibility was improved and shown to have a positive impact on skeletal muscle protein synthesis [31]. As such, we wanted to explore the effects of *V. faba*-derived hydrolysate on muscle mass including its potential as a food source for a nutritional intervention to address muscle atrophy.

The aim of this research was to validate the effects of natural peptide network_1 (NPN_1), a hydrolysate derived from *V. faba*, on protein synthesis, protein degradation, and inflammation in vitro. Followed by investigating the effects of NPN_1 (PeptiStrong™) ingestion on attenuating muscle waste in a disuse atrophy murine model.

## 2. Materials and Methods

### 2.1. Hydrolysate Production

We performed multiple hydrolyses on various different protein samples from different food sources, *Cicer arietinum* (Deltagen UK Ltd., UK; sample name: sh_DLBWW9), *Glycine max* (Shandong Sinoglory Health Food Co Ltd., China; sample name: sh_R79BKF), *Arthrospira platensis* (Iswari Superfood, Portugal; sample name: sh_JL39YJ), *Oryza sativa* (Shandong Jianyuan Bioengineering Co. Ltd., China; sample name: sh_5JQ01O), and *Vicia faba* (AGT Foods Europe, The Netherlands; sample name: NPN_1). All hydrolysates were prepared according to Rein et al. (2019) with some modifications [32]. In brief, protein powder from each source was homogenized in the solution and enzymatic hydrolysis was carried out with a food grade endoprotease under monitoring and control of enzyme-specific conditions, such as temperature and pH value (approximately pH 6). Following hydrolysis and enzyme inactivation by raising the temperature to 85 °C, higher than that reported by Rein et al. (2019), the solution was spray dried utilizing a standard spray drying process at air inlet temperatures above 160 °C. The spray dried powders were used for all assays and the murine atrophy model, they were prepared as follows: 150 mg of hydrolysate was weighed into a 15 mL tube. 5 mL of water was added to the tube and was vortexed until the powder was in the solution. The tube was centrifuged at 4000 rpm for 20 min and the supernatant was collected into a new tube. The supernatant was filtered through a 0.22 µM PES syringe filter (Fisherbrand, Thermo Fisher Scientific Inc., Canoga Park, CA, USA), homogenized, and protein content was determined using the BCA assay (Thermo Fisher Scientific Inc., Canoga Park, CA, USA).

### 2.2. Mass Spectrometry Analysis

The sample volume containing 5 mg of peptides is acidified with 0.2% formic acid (Sigma-Aldrich, St. Louis, MO, USA), desalted, and concentrated using Oasis HLB prime SPE cartridges (Waters Corporation, Milford, MA, USA). Eluates were lyophilized and resuspended in 100 μL of an Optima grade LC/MS water (Fisherbrand, Thermo Fisher Scientific Inc., Canoga Park, CA, USA). The peptide content is determined using the BCA assay. Aliquot containing 20 μg is resuspended in 0.1% TFA (Sigma-Aldrich, St. Louis, MO, USA), containing the Pierce™ peptide retention time calibration mixture, to the final concentration of 1 mg/mL (Thermo Fisher Scientific Inc., Canoga Park, CA, USA).

Samples were analyzed by the nano LC-MS/MS Dionex UltiMate 3000 coupled to a Thermo Fisher Q Exactive (Thermo Fisher Scientific Inc., Canoga Park, CA, USA) in a positive polarity mode. Peptides were loaded on a trapping column and eluted over a 25 cm analytical column PepMap RSLC C18 (Thermo Fisher Scientific Inc., Canoga Park, CA, USA) with a 1 h gradient at a flow rate of 300 mL min^−1^. The mass spectrometer was operated in a data-dependent mode, with MS and MS/MS performed in the orbitrap at 70,000 FWHM and 17,500 FWHM resolution, respectively. From the MS scan, the fifteen most intense ions were selected for MS/MS.

### 2.3. C2C12 Cell Culture

C2C12 cells are from a mouse skeletal muscle cell line (ECACC collection; Sigma-Aldrich, St Louis, MO, USA) and are a useful model to assess the myofilament function [16]. Cells were kept viable at 37 °C and 5% CO_2_. For preparation of the growth medium 1% L-glutamine solution, 1% penicillin-streptomycin (P4333, SIGMA), and a 10% sterile filtered foetal bovine serum previously heated at 55 °C for 30 min were added to 500 mL of 4.5 g/L glucose Dulbecco’s modified Eagle medium (DMEM) (BE12-614F, LONZA). To prepare the differentiation medium, 1% L-glutamine solution, 1% penicillin-streptomycin, and a 2% heat inactivated horse serum (26050-088, Gibco Life Technologies) were added to 500 mL of a 4.5 g/L glucose DMEM. Preparation of the starvation medium involved adding 1% L-glutamine solution and 1% penicillin-streptomycin to 500 mL of a 4.5 g/L glucose DMEM.

### 2.4. THP-1 Cell Culture

Human monocytic leukaemia (THP-1) cells (ECACC collection; Sigma-Aldrich, St Louis, MO, USA) were maintained in culture in the Roswell Park Memorial Institute medium (RPMI 1640, Lonza, Basel, Switzerland) supplemented with 1% L-glutamine, 10% heat-inactivated FBS, 1% penicillin–streptomycin, and a 10% sterile filtered foetal bovine serum previously heated at 55 °C for 30 min.

### 2.5. S6 Phosphorylation Assay

Cells were seeded 8000 cells/cm^2^ in 100 µL/well of the growth medium in a 96-well plate. Cells adhered and grew for 48 h at 37 °C, 5% CO_2_. Following this, the growth medium was removed and 100 μL of the differentiation medium was added to each well. Cells were allowed to differentiate for seven days at 37 °C, 5% CO_2_, by adding a fresh differentiation medium every day. The differentiation media was removed after seven days and 100 µl/well of the starvation medium was added to starve the cells for 3 h at 37 °C, 5% CO_2_. Following this, the starvation medium was removed and 100 μL/well of Hanks’ balanced salt solution (HBSS) was added and incubated at 37 °C, 5% CO_2_ to deprive cells of amino acids for 1 h.

Treatments were prepared by diluting the peptide network in HBSS to make up the desired concentration. All treatments were performed in triplicate. Phosphorylation of S6 (phospho-S6) was assessed by using the S6 In-Cell ELISA kit (Thermo Fisher Scientific, Waltham, MA, USA) according to the manufacturer’s instructions. C2C12 cells were treated with insulin (positive control, 0.58 µg/mL), NPN_1 (5–500 µg/mL) for 30 min following the starvation protocol and were compared to the untreated cells.

### 2.6. TNF-α Secretion Assay

THP-1 derived TNF released into the supernatants was assessed by using the TNF-α ELISA kit (BioLegend, San Diego, California, USA) according to the manufacturer’s instructions. To differentiate into macrophages, THP-1 cells were seeded (2 × 10^6^ well^−1^) in 6-well plates and treated with a 100 nM phorbol-12-myristate-13-acetate (PMA; Sigma-Aldrich, St Louis, MO, USA) for 72 h at 37 °C, 5% CO_2_. After incubation, non-attached cells were aspirated, and adherent cells were treated with NPN_1 (0.5–5 µg/mL) in duplicate or triplicate. Following incubation for 24 h, lipopolysaccharide (LPS) from Escherichia coli O127:B8 (Sigma-Aldrich, St Louis, MO, USA) was added to 100 ng/mL for 24 h at 37 °C, 5% CO_2_. Cell supernatants were collected, and THP-1 derived TNF released into supernatants was assessed by using the TNF-α ELISA kit (BioLegend, San Diego, California, USA) according to the manufacturer’s instructions.

### 2.7. RNA Isolation from C2C12 Cells and Real-Time QPCR

C2C12 cells were plated in 6-well plates and left to grow and differentiate at 37 °C, 5% CO_2_. The cells were subsequently starved for 24 h in a starvation media at 37 °C, 5% CO_2_. As per Menconi et al. (2008), to induce atrophy the cells were treated with 100 μM dexamethasone solubilized in a DMEM-LM (30030, BIOSCIENCES) supplemented 1% penicillin-streptomycin for 24 h at 37 °C, 5% CO_2_ [33]. Thirty minutes prior to the end of the atrophy induction, the cells were treated with NPN added on top of the dexamethasone treatment and incubated for 30 min at 37 °C, 5% CO_2_. Dilutions were calculated to get the desired concentration with the final volume of 2 mL/well. An equal volume of the treatment added to each well was first removed from the dexamethasone treatment without disturbing the cells. C2C12 cells were lysed with TRIzol (Invitrogen, Carlsbad, USA), and total RNA was extracted using the Purelink RNA mini kit (Invitrogen by Thermo Fisher Scientific) according to the manufacturer’s instructions. Total RNA (1 µg) was reverse transcribed to cDNA using the high-capacity cDNA reverse transcription kit (Thermo Fisher, Waltham, MA, USA). The quantitative PCR was performed using the TaqMan probe-based method, where mRNA expression was detected using a TaqMan fluorogenic gene expression probe (ABI Biosystems, CA, USA) for Trim63 (Mm01185221_m1) and Fbxo32 (Mm00499518_m1) for the experiment using C2C12. A master mix containing primer/probe and TaqMan^®^ gene expression master mix (ABI Biosystems, CA, USA) was added to 1 µL cDNA template. A final volume of 9 µL was pipetted, in duplicate, on a Roche Optical 96-well reaction plate and real-time PCR was performed on a Roche lightcycler 480 real-time PCR instrument.

The threshold cycle (Ct) for each well was calculated using the instrument software. Data analysis was based on the ΔΔCt method with raw data normalized by the B2M housekeeping gene (Mm00437762_m1) included on the plate. All gene expression was compared to dexamethasone, as dexamethasone was used to induce atrophy in C2C12 cells. NPN_1 was added subsequently to examine if it could attenuate the atrophy effect caused by the dexamethasone. Results are expressed as fold over control.

### 2.8. Toxicity Assay

An MTT assay was used to determine whether NPN_1 was toxic to the cells upon treatment. C2C12 cells were seeded in 96-well plates (1 × 10^5^ cells/well) and treated with NPN_1 (0.05–500 µg/mL). C2C12 cells were treated for 24 h before the well contents were removed and replaced with a MTT reagent (0.5 mg/mL). The cells were treated with a MTT reagent for 2 h at 37 °C, 95% humidity, and 5% CO_2_. MTT is a tetrazolium salt which is converted to formazan via succinate tetrazolium from the sarcoplasm reticulum in the mitochondria in viable cells only. The formazan salt is insoluble but becomes soluble in 100% DMSO. The MTT reagent is removed from the cells and replaced with 100% DMSO for 5 min while shaken on a plate shaker. The plate is read using a CLARIOstar BMGlabtech plate reader at 570 nm. The generated optical density values are converted to % with untreated cells set at 100%.

### 2.9. Disuse Murine Atrophy Study Protocol

This study was carried out with Melior Discovery, USA. Twelve-week old male C57b1/6J mice (*N* = 10/group) were randomly assigned to treatment groups based on bodyweight (10 days post-ring implantation). Ethical approval was granted by the International Association of Religious Freedom (IARF #:MLR-I15) and therefore, been performed in accordance with the ethical standards laid down by the Institutional Animal Care and Use Committee (IACUC).

The study consisted of five treatment groups (1) Healthy control (control weight bearing), (2) hindlimb unloaded (HU)-control vehicle (atrophy), (3) Bowman-Birk inhibitor (BBI; 113.3 mg/kg positive control), (4) casein (650 mg/kg; positive control), (5) NPN_1 (650 mg/kg). Briefly, prior to hindlimb unloading a tail ring was formed with a 2-0 sterile surgical steel wire that was passed through the 5th, 6th, or 7th inter-vertebral disc space and shaped into a ring from which the mice were suspended. The vertebral location for the tail-ring was selected to appropriately balance the animal body weight without interfering with defecation. The animals were suspended by a swivel harness attached at the top of the cage. The body of the animal was maintained at a 30° elevation such that only the forelimbs are to maintain contact with the cage floor. The animal could move and freely access food and water within the cage during this procedure. The height of the animal was checked daily and adjusted if necessary [34]. Mice were given seven days to acclimatize and condition, followed by 10–13 days of recovery time after tail-ring implantation, based on the IACUC guideline. The primary endpoint of this study was to assess the wet weight of the soleus muscle contained within the hindlimb of control and test mice directly after 19 days of hindlimb suspension. In addition, fixed muscle samples (5/group) were sent to CaresBio Laboratory LLC for immunofluorescence (IF) staining of Type I and Type IIa muscle fibre markers and image analysis. Soleus muscle tissue samples (10/group) were also sent to Cellomatics Biosciences LTD for gene expression analysis.

### 2.10. Dosing and Muscle Wet Mass

All mice were dosed with either NPN_1, BBI or Casein from day 1 to day 18. On day 19, animals were sacrificed by cervical dislocation; blood/plasma samples were collected, the soleus muscles were isolated and weighed using a digital platform balance. The wet muscle weights were normalized to body weights (mg/g). One side of the soleus muscle was snap frozen and the other side of the soleus muscle was fixed in 4% fresh PBS-buffered formaldehyde.

### 2.11. Immunofluorescence Analysis of Soleus

The collected soleus muscles were post-fixed in 4% fresh PBS-buffered formaldehyde. Five samples from each of the treatment groups were randomly selected for immunofluorescence analysis. Muscles were paraffin-embedded and sectioned. Immunofluorescence labeling was used to stain Type I and Type IIa muscle fibres. Image analysis was performed on representative regions of each sample for both staining labels. As samples were processed, sections (thickness, 8 µM) were cut and immunofluorescence staining was performed as previously described [35]. Briefly, slides were subjected to heat induced antigen retrieval in a citrate buffer (10 mM, pH 6.0) and were incubated overnight with primary antibodies ab11083 (dilutions 1:250) and ab91506 (dilutions 1:200), after blocking with a nonspecific antigenicity blocker. Both primary antibody concentrations were determined after optimization in the test slides. Corresponding fluorescent conjugated secondary antibodies (Alexa 594 and Alexa 488) were applied for 1 h at room temperature. 4,6-Diamidino-2-phenylindole (DAPI) were included with the secondary antibodies to visualize the nuclei.

### 2.12. Image Acquisition and Analysis

The slides were scanned using a customized, computer-controlled microscope (with xy-stage and z controller, a Zeiss microscope, Carl Zeiss GMBh, Jena, Germany) with X4, and X10 objectives. Images were analyzed using an image analysis software based on MATLAB (R2011b, MathWorks). The baseline for the scanning setup was done using the HU-control group. Image analysis algorithms were applied to the images generated from microscopic slides of tissues stained with secondary antibody controls to generate the background score. The control/baseline was used to generate the algorithm to differentiate between the signals and signal-to-noise ratio which was applied to all images. Each marker was quantified by the single channel-based analysis. Automatic background subtraction was performed. Intensity scores for all the markers were then calculated that correspond to the average signal intensity divided by a locale area. Significant differences in relative areas stained and mean specific intensity for the stains of different groups in mouse muscle tissue were calculated. Raw data is presented, and no normalization was performed.

### 2.13. RNA Isolation from Mouse Soleus Tissue and Gene Arrays

Gene expression analysis was performed on soleus tissue samples. 300 µL of a homogenization solution with 3 µl Proteinase K (Quantigene Plex Assay, Invitrogen) were added to 10 mg of frozen tissue to prepare concentrated lysates. One 5-mm stainless steel bead was added to the tubes and placed in a Bullet Blender^®^ homogenizer. The tissue was then homogenized for 2 min at Speed 10. The tubes were allowed to cool at room temperature and the process was repeated until no visible particles remained. The tissue lysates were then incubated at 65 °C for 30 min, followed by centrifugation at 16,000× *g* for 15 min and the supernatant was used immediately for the assay kit (as per the manufacturer’s guidelines). The net mean fluorescent intensity (MFI) was obtained from the Luminex for all the genes.

### 2.14. Statistics

All statistical analyses were performed using the statistical computing software R [36]. For S6 phosphorylation and TNF-α secretion assays, significant differences from untreated controls were determined by one-way ANOVA followed by a Dunnett’s test. Data is presented as a percentage of untreated controls (the mean ± SEM of at least three independent experiments). For Trim63 and Fbxo32 gene expression, data were analyzed with two-way ANOVA and Tukey’s multiple comparisons test. For preclinical data, significant differences from the control vehicle were determined using normalized wet weights of the soleus muscle which were represented as the mean ± SEM and analyzed by one-way ANOVA followed by multiple comparisons tests as applicable. Type I and Type IIa fibres of the soleus muscle were analyzed by the unpaired T-test with Welch’s correction as applicable. For all analyses, *p*-value < 0.05 was considered significant. Graphs were generated using the ‘ggplot2′ R package [37].

## 3. Results

### 3.1. Bioactivity Screening

**Figure 1:**
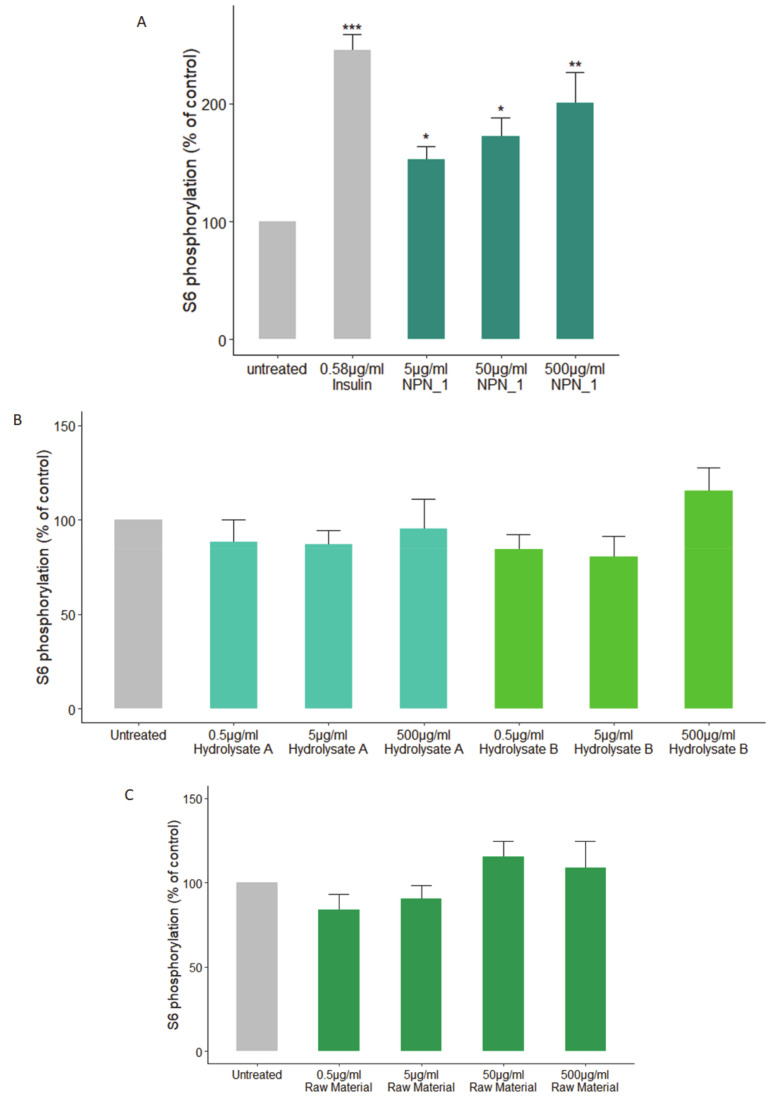
Effect of the natural peptide network_1 (NPN_1) treatment on S6 phosphorylation. C2C12 cells were treated with (**a**) NPN_1 (5–500 μg/mL), (**b**) hydrolysate A, hydrolysate B or (**c**) raw material (5–500 μg/mL) for 30 min following a starvation protocol and compared to untreated cells (control) and expressed as the % of controls (one-way ANOVA analysis; * *p* < 0.05; ** *p* < 0.01; *** *p* < 0.001; at least three independent replicates).

To examine the possibility of identifying a hydrolysate with the potential to address skeletal muscle wasting we used phosphorylation of S6 as an initial screen. Using a number of natural sources, C2C12 mouse skeletal muscle cells were challenged with different concentrations for 30 min. Following a starvation protocol, NPN_1 (*V. faba* hydrolysate) alone, significantly increased the phosphorylation of S6 at all concentrations at 30 min compared to the untreated C2C12 muscle cells (Figure 1A). Treatment with alternate sources did not exhibit any significant effects on S6 phosphorylation (see Supplemental data Figure S1). Focusing on *V. faba*, two hydrolysates were produced using different buffers in the preparation for hydrolysis than described for NPN_1. Hydrolysate A was produced in a neutral solution and hydrolysate B was produced using a saline solution. Other proteolytic hydrolysates that were produced from *V. faba* showed lower or no efficacy when tested (Figure 1B). Additionally, the effect of unhydrolyzed *V. faba* on S6 phosphorylation was examined using C2C12 muscle cells, the raw material had no effect on S6 phosphorylation (Figure 1C). When combined, these results suggest a specific peptide network driven activity.

**Figure 2:**
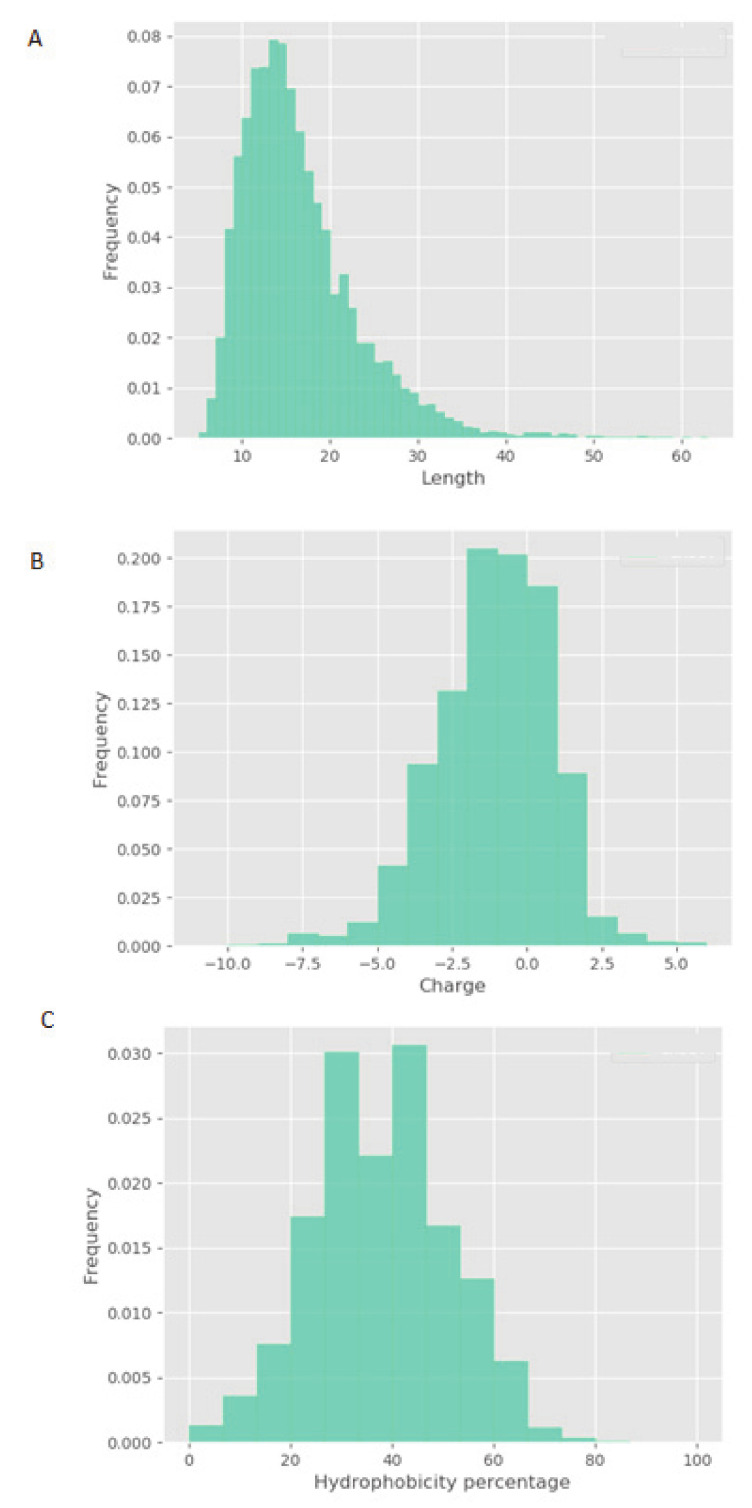
Physicochemical properties of the NPN_1 peptide profile as determined by LC-MS/MS. Histogram representation of the NPN_1 peptide distribution according to (**a**) length, (**b**) charge, and (**c**) percentage hydrophobicity; peptide counts are displayed on the y-axis.

LC-MS/MS was used to characterize NPN_1. Figure 2 highlights the physicochemical properties of peptide profiles contained within NPN_1, including relative hydrophobicity, sequence length, and charge of peptides. Most of the NPN_1′ constituent peptides fall between 5 and 20 amino acids in length (Figure 2A) and feature a global charge range from −5 to +1, where a net charge of -1 occurs most frequently (Figure 2B). Finally, the majority of the peptides within NPN_1 contain approximately 35–45% of hydrophobic residues (Figure 2C).

**Figure 3:**
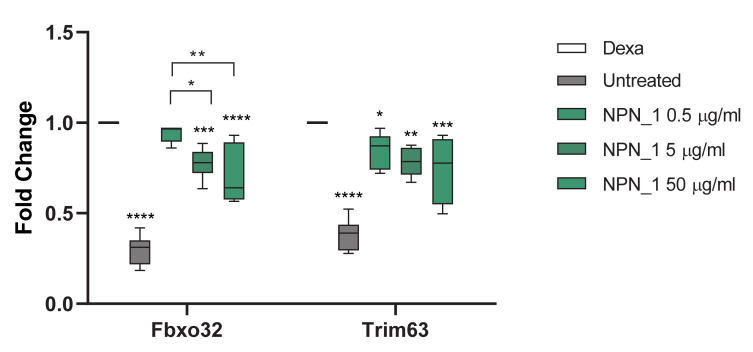
Effect of NPN_1 on atrophy related gene expression. The PCR analysis was carried out on atrophy induced C2C12 cells showing the effect of NPN_1 on Fbxo32 and Trim63 gene expression. Cells were treated with dexamethasone (Dexa; 0.3 μg/mL) for 24 h, 30 min prior to the end of the dexamethasone treatment, NPN_1 (0.5–50 μg/mL) was added. Untreated cells were not treated with dexamethasone or NPN_1 (two-way ANOVA with Tukey’s multiple comparisons test; * *p* < 0.05; ** *p* < 0.01; *** *p* < 0.001; **** *p* < 0.0001; at least four independent replicates).

The effect of NPN_1 on muscle atrophy in vitro was also assessed (Figure 3). Gene expression levels of genes related to protein degradation and muscle loss were measured, to investigate if NPN_1 could reduce muscle loss. Dexamethasone was used to induce skeletal muscle atrophy, interestingly treatment with NPN_1 significantly attenuated expression of genes related to atrophy similar to that seen in cells untreated with dexamethasone (untreated). Fbxo32 encodes Atrogin-1 and Trim63 encodes Murf-1. Trim63 gene expression was significantly reduced following the NPN_1 treatment in comparison to dexamethasone (Dexa) treated cells (*p* < 0.05) (Figure 3). Fbxo32 gene expression was significantly reduced in a dose-dependent manner following increased concentrations of NPN_1 treatment (Figure 3). Additionally, C2C12 viability, in response to the NPN_1 treatment, was assessed by an MTT assay, where no adverse effect was reported up to 500 µg/mL (see Supplemental data Figure S2).

**Figure 4:**
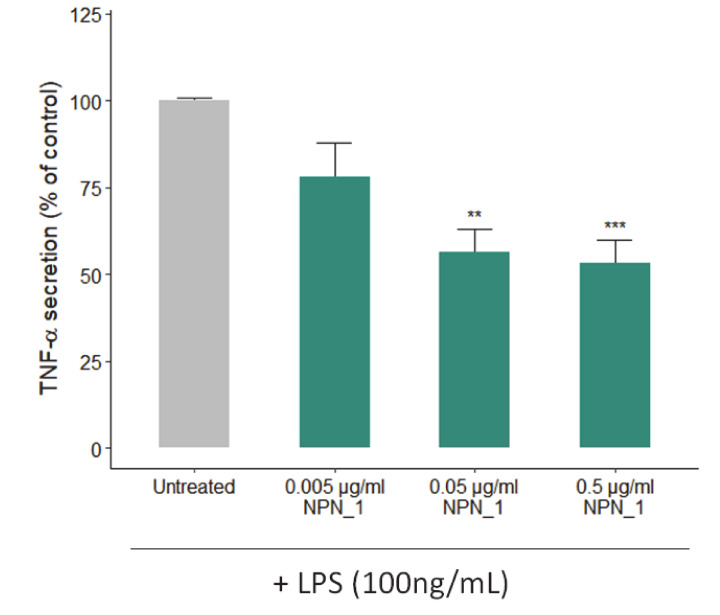
Effect of NPN_1 on TNF-α secretion in THP-1 differentiated macrophages. THP-1 macrophages were treated with NPN_1 (0.005–0.5 µg/mL) for 24 h before treating with 100 ng/mL of LPS for 24 h. The secretion of TNF-α was quantified by ELISA (one-way ANOVA analysis; ** *p* < 0.01; *** *p* < 0.001; at least three independent replicates).

To further validate the NPN_1 effect on muscle health, the effects of NPN_1 on TNF-α secretion were tested in vitro using THP-1 human monocytes. For that purpose, cells were pre-treated with NPN_1 (Figure 4) for 24 h before adding lipopolysaccharide (LPS) for another 24 h. Our results show that NPN_1 significantly prevented the LPS-induced TNF-α protein secretion by THP-1 monocytes at higher concentrations (0.05 and 0.5 µg/mL).

### 3.2. Preclinical Efficacy of NPN_1

**Figure 5:**
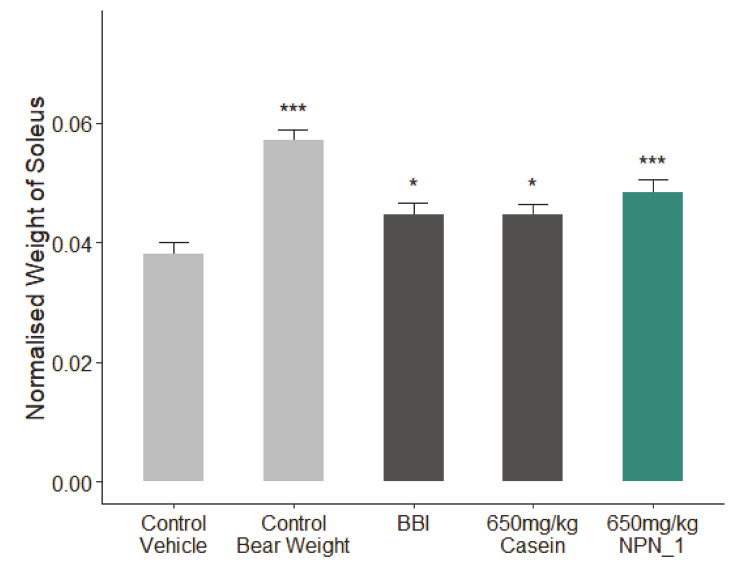
The effect of NPN_1 on soleus muscle mass following hindlimb unloading. C57BL/6 mice were treated with the Bowman-Birk inhibitor (BBI) (113.3 mg/kg per day), casein (650 mg/kg per day) or NPN_1 (650 mg/kg per day) over the course of 18 days (one-way ANOVA analysis; * *p* < 0.05; *** *p* < 0.001; N = 10/group).

The potential muscle health benefits of NPN_1 was tested in vivo in a disuse-induced muscle atrophy mouse trial. In this study, we administrated NPN_1 to a group of hindlimb unloaded mice for 18 days. On day 19, wet weights of both sides of the soleus muscle were weighed immediately after tissue collection. The bodyweights were used to normalize the soleus muscle wet weights changes. Compared to weight bearing control animals, control vehicle animals showed significant reduction in normalized soleus muscle wet weight (Figure 5). On average, control bear weight animals exhibited 49.9% more soleus muscle mass than the control vehicle group. Additionally, the normalized soleus muscle weights of groups of casein (15.8%), BBI (17.3%), and the NPN_1 (27.1%) were significantly increased. Compared to the muscle atrophy experienced in the control vehicle (HU) animals, NPN_1 attenuated muscle loss in the soleus by almost 50%. This result was observed after just 18 days of treatment. This effect was seen at 650 mg/kg of treatment of NPN_1, indicating the potential for a low human equivalent dose (2.8 mg/day) for future human efficacy studies [38]. As casein has been shown in the past to be associated with muscle protein synthesis [39], it was chosen as a comparator (protein content of >81%). A second comparator, BBI, a drug that has also been shown to attenuate muscle atrophy, was also used in this study [34].

**Figure 6:**
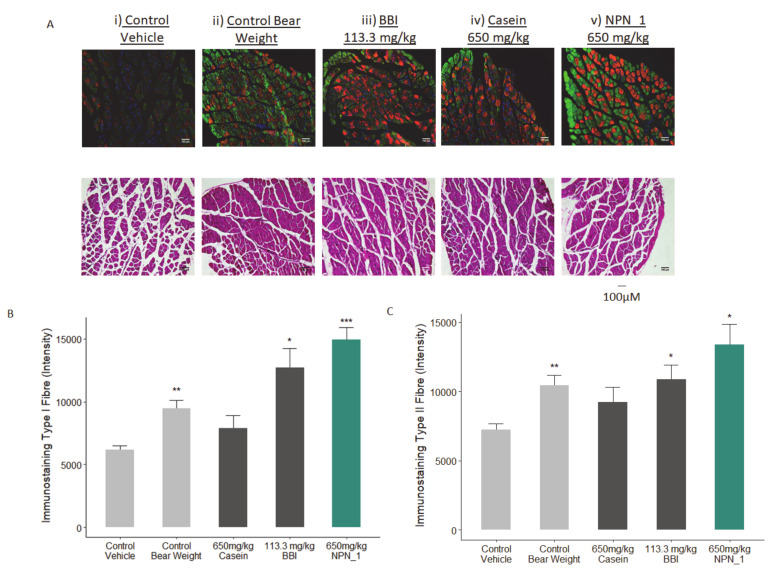
The effect of NPN_1 on Type-I and Type IIa muscle fibre expression. (**A**) Skeletal muscle immunostaining on Type-I (Red) and Type IIa (Green) muscle fibre expression and hematoxylin and eosin (H&E) staining demonstrating the effect of NPN_1. Quantification of the effect of treatment on Type I (**B**) and Type IIa (**C**) fibre expression (* *p* < 0.05; ** *p* < 0.01; *** *p* < 0.001; N = 5).

To assess the morphological protective effect of NPN_1 on muscle atrophy/waste, H&E staining on the soleus muscle was carried out, similar to Noh et al. (2015) [40]. As shown in Figure 6, muscle fibres in the control bear weight group were in closer contact, showing a more uniform disposition than in the control vehicle group, where immobilization-induced atrophy caused damage to muscle fibre distribution and fibre bundles, resulting in an atrophic pattern. These atrophic changes were ameliorated by the NPN_1 treatment, suggesting that our ingredient effectively prevented immobilization-induced muscle damage (Figure 6A). We also analyzed Type I and Type IIa muscle fibres as these are essential for endurance and rapid movement, respectively. The Type I muscle fibre also known as the slow-twitch fibre, supports long distance endurance i.e. marathons, while Type IIa known as fast-twitch supports fast and powerful movements i.e. weightlifting. The effect on Type I and Type IIa muscle fibre expression was explored following treatment with the positive control BBI, casein or NPN_1 over 18 days of administration. Casein treatment did not significantly affect Type I and Type IIa fibre expression. A significantly greater intensity of immunofluorescence was observed in NPN_1-treated mice when comparing both Type I and Type II fibres to control vehicle samples (Figure 6B,C). This strengthens claims regarding NPN_1’s protective effects against muscle atrophy on both muscle bundles and individual fibre structures in H&E staining investigations, as seen in Figure 6A. Compared to the control vehicle, BBI showed a similar profile to that of NPN_1, however, the intensity levels and statistical significance associated with BBI treatment were less than that recorded in NPN_1 samples for Type I fibre immunostaining (Figure 6B).

RNA was extracted from collected soleus tissue samples from control vehicle and NPN_1 treated animals (N = 10/group). A focused panel of 11 genes was investigated to support specific myogenesis responses, which were of a translational nature to the clinic, and to rule in or out any secondary mechanism of action that maybe contributing to the observed muscle mass efficacy (Table 1). The outcomes suggest that the confirmed myogenesis (mTOR, MYF5) and mitochondrial biogenesis (TFAM, ESRRA) responses are occurring without affecting recognized antioxidant or fatty acid oxidation pathways, due to the lack of an effect on both NRF genes and CPT1B, respectively. Interestingly, four oncogenic genes (also associated with muscle regeneration) were unaffected or downregulated following 18 days of treatment and provides initial confidence around the safety aspects of this natural product.

## 4. Discussion

In this study, we present evidence validating the use of a hydrolysate resulting from a proteolytic hydrolyses process of *V. faba* in addressing a critical unmet need, muscle wasting.

S6K1-phosphorylated S6 ribosomal protein is a well-known mTORC1 downstream marker in the canonical Akt/mTOR pathway, which is the primary axis that controls protein synthesis in the cell [7]. Phosphorylation of S6 correlates with an increase of the translation of mRNA transcripts that encode other ribosomal proteins and elongation factors necessary to initiate the translation process that leads to muscle protein synthesis [41,42]. We observed a significant increase in S6 phosphorylation following the NPN_1 treatment. Alternative food-derived hydrolysates and other proteolytic hydrolyses of *V. faba* showed little or no efficacy in vitro. These results highlight that the phosphorylation activity observed is, most likely, peptide driven. Although physicochemical properties of constitutive peptides within NPN_1 are shown, a further characterization of NPN_1 is required to understand which peptides carry this activity and more importantly to further understand if these peptides resist proteolytic degradation caused by the digestive system and whether they can be bioavailable (work currently in preparation).

Treatment with NPN_1 significantly reduced the expression of Fbxo32 and Trim63. Fbxo32 is highly expressed during muscle atrophy, whereas mice deficient in this gene were found to be resistant to atrophy [9]. Fbxo32 encodes Atrogin-1 whose levels are elevated in the skeletal muscle during fasting, prior to atrophy [9]. Thus, this protein is a potential target for the treatment of muscle atrophy. Atrogin-1 is an F-box protein or Ub-protein ligase (E3) expressed in a tissue specific manner and appears to be a critical component in the enhanced proteolysis leading to muscle atrophy in diverse diseases [43]. Muscle specific Trim63 controls the proteasomal degradation of muscle proteins under amino acid starvation, where muscle protein is broken down to supply other organs with amino acids [44].

It has been previously demonstrated in vitro and clinically, that conditions and diseases like inactivity, aging or cancer can cause loss of strength, frailty, high muscle protein breakdown rates, and different degrees of inflammation [45,46,47]. Of note, we observed a significant reduction of TNF-α secretion, as the immune system and its regulation has been found to be a key mediator that under these conditions induces muscle atrophy [45]. Muscle exposure to TNF-α increases protein degradation and muscle loss in a process regulated by NF-κB [15,48]. This loss of muscle mass has been reported to occur via the ubiquitin protein system as TNF-α administration upregulates the ubiquitin conjugating activity and proteolytic degradation markers [49,50]. Importantly, TNF-α could also impact muscle function and strength by activating caspase-3 that degrades the actin and myosin contractile filaments [51].

Our preclinical work in mice validated our in vitro findings. Additionally, NPN_1 exceeds the efficacy of both casein and BBI in soleus muscle mass weight and in skeletal muscle fibre expression. These in vivo results have shown the muscle health benefits of NPN_1 by ameliorating the devastating effects of disuse-induced muscle atrophy on muscle mass and at doses indicating low human dose equivalency. There are several signaling mechanisms responsible for skeletal muscle atrophy following mechanical unloading. Previous research on disuse rodent models found a reduced phosphorylation state of S6K1 in the soleus and medial gastrocnemius muscles due to an Akt/mTORC1 pathway attenuation caused by the impairment of IGF-1 signaling [8,52]. The reduction of Akt phosphorylation also upregulates the atrogenes MurF-1 and MAFbx expression which impacts positively on muscle atrophy development. Oxidative stress during prolonged periods of disuse has also been reported to affect protein synthesis and breakdown pathways. There are several factors that could lead to this stress, for example, calcium overload or inflammation through NF-κB [53]. Based on the significance of our in vitro and in vivo data we can suggest that the multiple activity of bioactive peptides within NPN_1 are major players contributing to muscle health effects. The attenuation of soleus wet weight loss by NPN_1 is at least, in part, likely due to its anti-TNF-α activity which eventually lowers the inflammation state that promotes muscle atrophy. This anti-inflammatory activity happens during induction of protein synthesis through S6 phosphorylation, positioning NPN_1 as a promising peptide-based intervention to address muscle waste.

During atrophy, the link between mitochondrial mediated oxidative stress and insulin resistance represents a mechanism by which impaired mitochondrial activity cause poor recovery of muscle mass. Transcriptional regulators of mitochondrial biogenesis and function, ERRα (ESRRA), and mitochondrial transcription factor A (TFAM) are also expected to be inhibited during unloading [54]. Considering that slow-twitch (Type 1) skeletal muscle fibres, that are mainly found in soleus muscle, have markedly greater mitochondrial content than fast-twitch (Type 2) fibres, suggesting that the ESRRA and TFAM gene upregulation seen in our in vivo gene expression results are positive supporting markers contributing to muscle recovery and regeneration. Skeletal muscle regeneration is a complex and extremely efficient process that enables muscles to retain effective regenerative capabilities even after several injuries. The PPP3CA gene codes for calcineurin, a serine/threonine phosphatase that is activated by sustained increased levels of intracellular Ca^2+^. Calcineurin participates in a variety of processes including myoblast recruitment, myotube differentiation, fibre type specification, and recovery from muscle injury and dystrophic muscle damage. It has been shown that calcineurin signaling maintains muscle size, but it is suppressed under atrophy conditions, especially in slow muscle fibres present in the soleus [55]. In our gene expression array, we showed that PPP3CA was significantly upregulated by the NPN_1 treatment.

Further investigations to unveil an additional myogenesis mechanism of action would further explain the overall activity of NPN_1.

## 5. Conclusions

The intricate nature of muscle wasting results from the complex overlap of different pathways all contributing to muscle metabolism. A solution to this problem cannot be a one target approach but needs to influence multiple targets underlining atrophy. As such, a mixture of peptides with different mechanisms is one such potential approach. Here, by targeting multiple mechanisms such as inflammation, by reducing TNF-a; muscle synthesis, by augmenting S6 phosphorylation; and the downregulation of genes related to muscle atrophy, an overall efficacy for attenuating muscle atrophy was achieved. To date, we are not aware of any validated nutritional interventions that can preserve muscle mass, therefore, a further human study is warranted and an assessment of NPN_1 as a functional ingredient has progressed to the clinic and is currently underway in a double-blind placebo-controlled study.

## Figures and Tables

**Table 1:** Fold regulated gene expression of NPN_1.

Gene	Fold Change		
mTOR	4.19		
ESRRA	3.15		
MYF5	3.06		
TFAM	3.44		
WNT1		−3.17	
IGF1R		−3.42	
MYC		−3.44	
CPT1B			NC
NRF1			NC
NRF2			NC
KRAS			NC

## Supplementary Materials

The following are available online at https://www.mdpi.com/2072-6643/12/8/2274/s1. Figure S1: Effect of alternative food-derived hydrolysate treatment on S6 phosphorylation; Figure S2: MTT assay on C2C12 cells treated NPN_1.
