# Supplementary material for: Preclinical Evaluation of a Food-Derived Functional Ingredient to Address Skeletal Muscle Atrophy
Source: Nutrients. 2020 Jul 29;12(8):2274. doi: 10.3390/nu12082274 (PMC7469066; doi:10.3390/nu12082274)
Supplement: Supplementary file 1 [file nutrients-12-02274-s001.pdf]

Supplemental Data

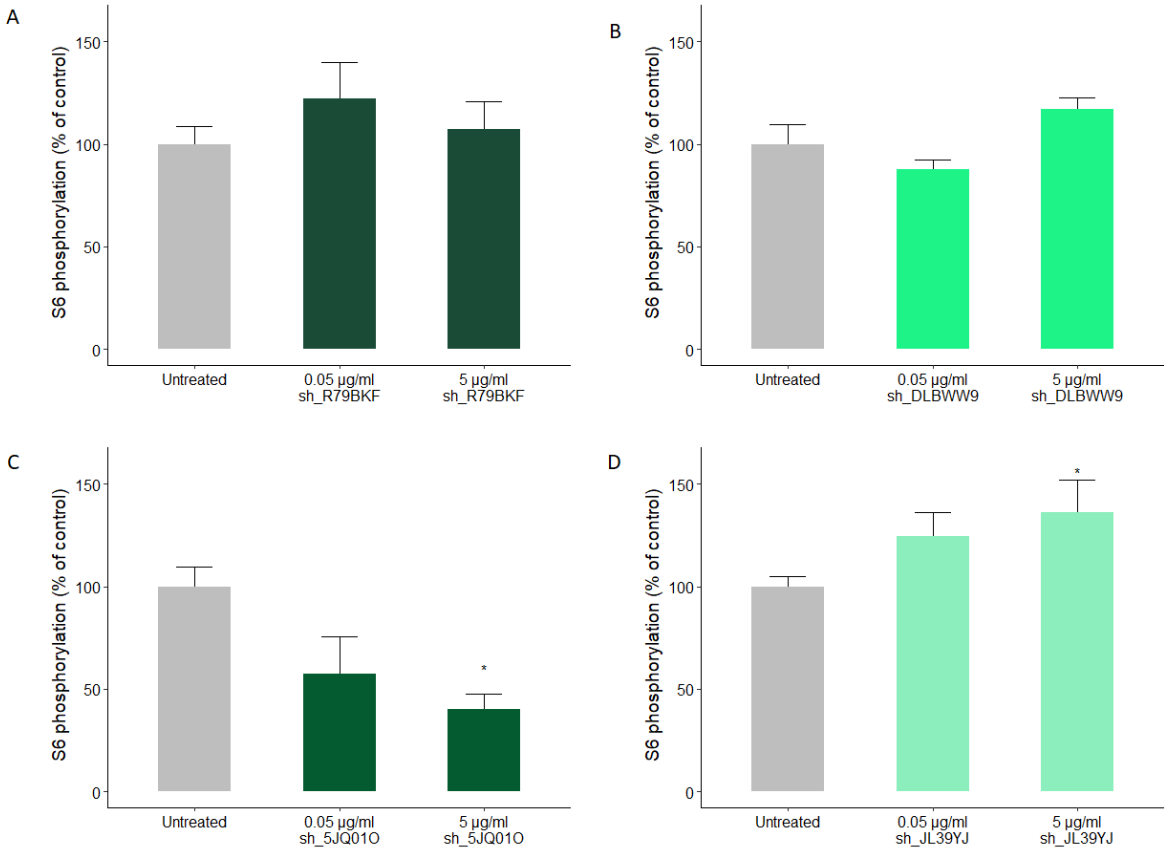

Figure S1. Effect of alternative food-derived hydrolysate treatment on S6 phosphorylation. C2C12 cells were treated with (**a**) sh_R79BKF (N=3), (**b**) sh_DLBWW9 (N=1), (**c**) sh5JQ01O (N=3) or (**d**) sh_JL39YJ (N=1; 0.5 µg/mL and 5 µg/mL) for 30 mins following a starvation protocol and compared to untreated cells. (One-way ANOVA analysis; *p<0.05 **p<0.01 ***p<0.001).

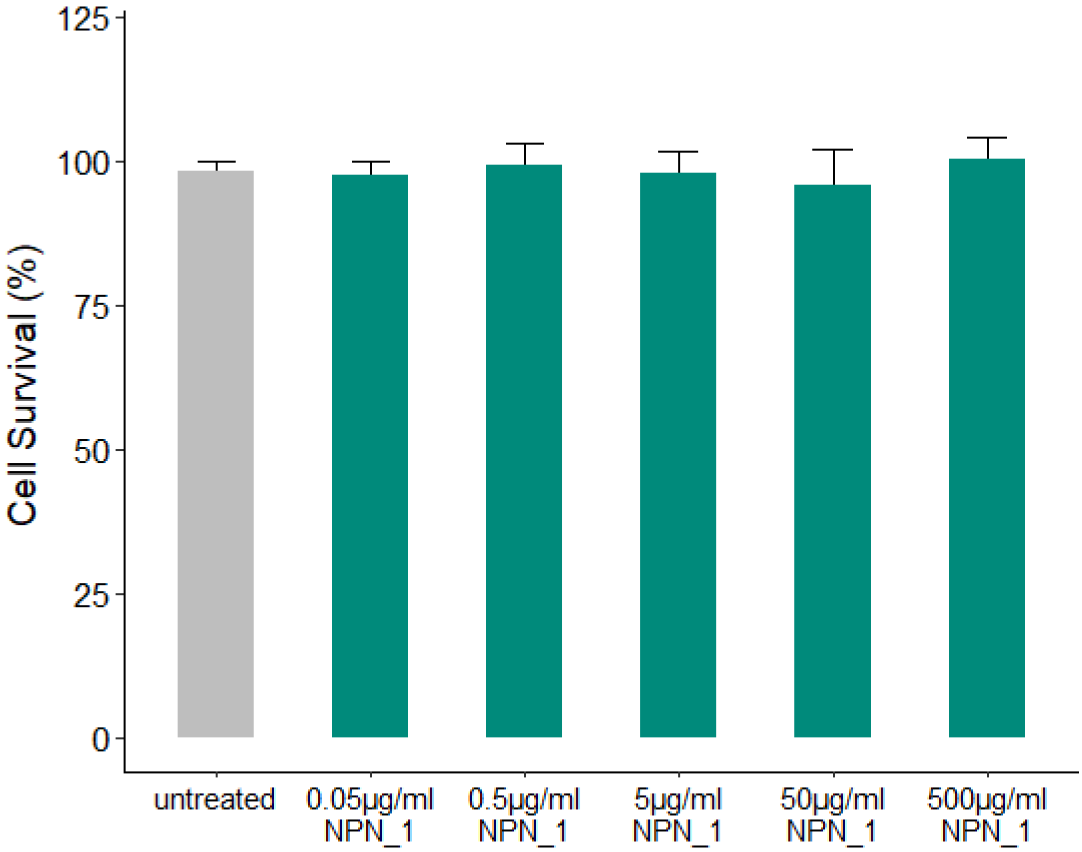

Figure S2. MTT assay on C2C12 cells treated NPN_1. Percentage viability of C2C12 cells following 24 h incubation with increasing concentrations of NPN_1. Data presented are the mean ± SEM of at least 2 independent experiments carried out in triplicate. (*$p<0.05$ **$p<0.01$ ***$p<0.001$).
